# Identification of key genes and pathways in discoid lupus skin via bioinformatics analysis

**DOI:** 10.1097/MD.0000000000025433

**Published:** 2021-04-23

**Authors:** Qian Dong, Kang Chen, Jinye Xie, Hui Han, Yanping Feng, Jianqiang Lu, Weijia Wang

**Affiliations:** Department of Laboratory Medicine, Zhongshan Hospital of Sun Yat-sen University, Zhongshan, Guangdong, China.

**Keywords:** bioinformatics, differential gene, discoid lupus erythematosus, Kyoto Encyclopedia of Genes and Genomes pathway

## Abstract

Discoid lupus erythematosus (DLE) is the most common skin manifestation of lupus; however, the molecular mechanisms underlying DLE remain unknown. Therefore, we aimed to identify key differentially expressed genes (DEGs) in discoid lupus skin and investigate their potential pathways.

To identify candidate genes involved in the occurrence and development of the disease, we downloaded the microarray datasets GSE52471 and GSE72535 from the Gene Expression Database (GEO). DEGs between discoid lupus skin and normal controls were selected using the GEO2R tool and Venn diagram software (http://bioinformatics.psb.ugent.be/webtools/Venn/). The Database for Annotation, Visualization, and Integrated Discovery (DAVID), Enrichr, and Cytoscape ClueGo were used to analyze the Kyoto Encyclopedia of Gene and Genome pathways and gene ontology. Protein-protein interactions (PPIs) of these DEGs were further assessed using the Search Tool for the Retrieval Interacting Genes version 10.0.

Seventy three DEGs were co-expressed in both datasets. DEGs were predominantly upregulated in receptor signaling pathways of the immune response. In the PPI network, 69 upregulated genes were selected. Furthermore, 4 genes (CXCL10, ISG15, IFIH1, and IRF7) were found to be significantly upregulated in the RIG-I-like receptor signaling pathway, from analysis of Enrichr and Cytoscape ClueGo.

The results of this study may provide new insights into the potential molecular mechanisms of DLE. However, further experimentation is required to confirm these findings.

## 1 Introduction

Systemic lupus erythematosus (SLE) is a chronic autoimmune disease affecting numerous organ systems with cutaneous manifestations in over half of affected individuals.^[1]^ Certain cutaneous lupus erythematosus (CLE) subtypes can also occur in the absence of systemic diseases.^[2]^

Discoid lupus erythematosus (DLE), the most common chronic cutaneous lupus subtype,^[3,4]^ is a photosensitive, disfiguring skin disease marked by scaly erythematous papules located most commonly on the face, scalp, and neck,^[2,5]^ leading to prominent scarring that might have a high impact on the quality of life of patients.^[6]^ First-line therapies for DLE include antimalarial agents and topical steroids, together with sun protection. In many cases, the available systemic agents are unable to adequately control this disease. A critical need, therefore, exists for the development of a targeted therapeutic agent with a favorable side effect profile.

Due to the limited understanding of DLE pathogenesis, effective treatment options are limited. The data on gene expression profiles have greatly increased in recent years and it has, by taking advantage of bioinformatics methods, become a hot topic of new research to interrogate the available data.

To identify signaling pathways and cellular signatures for possible treatment targeting, we profiled the transcriptome of DLE skin.

## 2 Methods

### 2.1 Microarray data information

We obtained the gene expression profiles of GSE52471 and GSE72535 in discoid lupus skin and normal controls from NCBI-GEO (https://www.ncbi.nlm.nih.gov/pubmed), a free public database of microarray/gene profiles. Ethical approval was not necessary for this study because public datasets were analyzed. Microarray data of GSE52471 and GSE72535 were all on GPL571 Platforms ([HG-U133A_2] Affymetrix Human Genome U133 2.0 Array), which included 11 DLE skin lesion samples and 3 normal skin samples, and 9 DLE lesion skin samples and 8 normal skin samples, respectively.

### 2.2 Data processing of differentially expressed genes (DEGs)

DEGs between DLE lesions and normal skin were selected from the GEO2R online tool (http://www.ncbi.nlm.nih.gov/geo/geo2r/).^[7]^ We identified the DEGs with |log fold-change (FC)| > 2 and adjusted *P*-value <.05, and then checked using Venn software online to detect common DEGs between the 2 datasets. DEGs with log FC < –2 were considered as downregulated genes, while DEGs with log FC > 2 were considered as up-regulated genes.

### 2.3 Gene ontology and pathway enrichment analysis

Gene ontology analysis (GO) is a commonly used approach to define genes and their RNA or protein products and to identify unique biological properties of high-throughput transcriptome or genome data.^[8]^ Kyoto Encyclopedia of Genes and Genomes (KEGG) is a collection of databases of genomes, diseases, biological pathways, drugs, and chemical materials.^[9]^

Database for Annotation, Visualization, and Integrated Discovery (DAVID) software (https://david.ncifcrf.gov/) is an online bioinformatics tool designed to identify the functions of large numbers of genes or proteins^[10]^ and can be used to visualize the enrichment of DEGs in biological process (BP), molecular function (MF), and cell component (CC) pathways (*P* < .05).

### 2.4 Protein-protein interactions (PPI) network and module analysis

The potential correlation between these DEGs (maximum number of interactors = 0 and confidence score ≥0.4) was determined using the online PPI information evaluation tool STRING (Search Tool for the Retrieval of Interacting Genes: https://string-db.org/),^[11]^ in Cytoscape.^[12]^ In addition, modules of the PPI network (degree cutoff = 2, max. depth = 100, k-core = 2, and node score cutoff = 0.2) were checked using the Molecular Complex Detection plugin (MCODE) app in Cytoscape.

### 2.5 Pathway Analyses

KEGG analyses were performed using Enrichr (http://amp.pharm.mssm.edu/Enrichr/)^[13,14]^ and Cytoscape Go bioinformatics tool.^[15,16]^ Pathway enrichment analyses were based on a cut-off value of *P* < .05.

## 3 Results

### 3.1 Identification of DEGs in DLE

**Figure 1:**
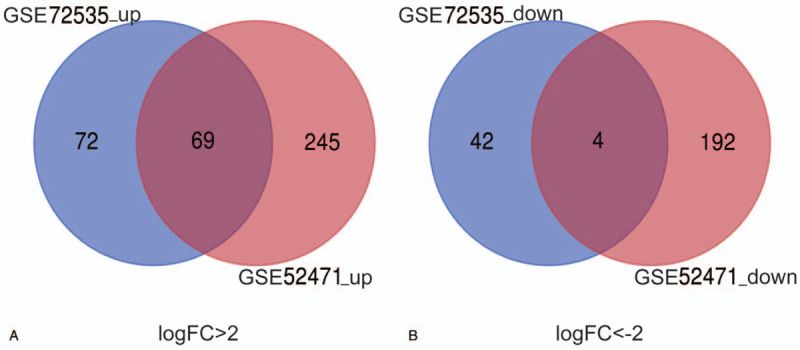
Total of 216 common DEGs in the 2 datasets (GSE52471 and GSE72535) identified through Venn diagram software. A, Sixty nine DEGs were upregulated in 2 datasets (logFC > 2). B, Four DEGs were downregulated in 2 datasets (logFC < –2). DEGs = differentially expressed genes.

We obtained the gene expression profiles of GSE52471 and GSE72535 in discoid lupus skin and normal controls from NCBI-GEO. To identify the DEGs from these 2 groups (DLE and normal controls), we conducted GEO2R web-server analysis to calculate the *P*-values and |logFC| values.^[17]^ Using GEO2R online tools, we identified 437 and 114 DEGs, respectively. We identified common DEGs in the 2 datasets using Venn diagram software and showed a total of 73 common DEGs, including 4 downregulated genes (logFC < –2) and 69 upregulated genes (logFC > 2), in DLE (Table 1 and Fig. 1).

**Table 1:** All 73 common differentially expressed genes (DEGs) were detected from two profile datasets in DLE lesions compared to normal skin.

DEGs	Genes Name
Up-regulated	IL7R IFI35 CXCL10 HERC5 IDO1 STAT2 CCL5 OAS3 OAS1 RSAD2 MX1 CXCL13 WARS TRIM22 LTB LGALS3BP BST2 IRF7 CFB SP110 IFI6 HERC6 CD8A XAF1 IFIH1 ADAMDEC1 GZMA CD2 AIM2 IRF8 USP18 TDO2 SAMD9 TLR7 KRT6B IFI44L IFIT1 PLAC8 LAMP3 CCL8 GBP1 OAS2 CD3D KRT16 CXCL9 FCMR GZMB ZBP1 PI3 LAG3 ISG20 OASL CXCL11 C1QB STAT1 TYMP JCHAIN RTP4 IFI44 CD48 GZMK CCR7 ISG15 MX2 IFIT3 MMP9 GNLY NKG7 IFI27
Down-regulated	PON3 FABP7 COCH HSD11B1

### 3.2 DEGs gene ontology and KEGG pathway analysis in DLE

All 73 DEGs were analyzed using DAVID software and the GO analysis results indicated that for biological processes (BP), DEGs were upregulated in immune response, innate immune response, inflammatory response, response to interferons (alpha and beta), and chemokine-mediated signaling pathway regulation of interleukin-12 production; for molecular function (MF), DEGs were upregulated in chemokine activity, double-stranded RNA binding, CXCR chemokine receptor binding, nucleotidyltransferase activity, regulatory region DNA binding, and serine-type endopeptidase activity; and for GO cell components (CC), DEGs were significantly upregulated on the external side of the plasma membrane, cytoplasm, extracellular space, and blood microparticles (Table 2).

**Table 2:** Gene ontology analysis of differentially expressed genes in DLE.

Category	Term	Count	%	*P*-value	FDR
GOTERM_BP_DIRECT	GO:0006955∼immune response	13	16.34	1.11E–11	1.48E–08
GOTERM_BP_DIRECT	GO:0070098∼chemokine-mediated signaling pathway	6	7.54	1.03E–06	0.00138
GOTERM_BP_DIRECT	GO:0006954∼inflammatory response	9	11.31	2.70E–06	0.003596
GOTERM_BP_DIRECT	GO:0045087∼innate immune response	8	10.05	1.17E–05	0.0156
GOTERM_BP_DIRECT	GO:0035455∼responseto interferon-alpha	3	3.77	.00102	1.352729
GOTERM_CC_DIRECT	GO:0009897∼external side of plasma membrane	8	10.05	2.80E–06	0.002757
GOTERM_CC_DIRECT	GO:0005737∼cytoplasm	19	23.88	.011213	10.49805
GOTERM_CC_DIRECT	GO:0005615∼extracellular space	9	11.31	.022916	20.389
GOTERM_CC_DIRECT	GO:0072562∼blood microparticle	3	3.77	.042296	34.62742
GOTERM_MF_DIRECT	GO:0008009∼chemokine activity	6	7.54	8.76E–07	9.22E–04
GOTERM_MF_DIRECT	GO:0003725∼double-stranded RNA binding	6	7.54	2.17E–06	0.002284
GOTERM_MF_DIRECT	GO:0045236∼CXCR chemokine receptor binding	3	3.77	.001204	1.259238
GOTERM_MF_DIRECT	GO:0016779∼nucleotidyltransferase activity	3	3.77	.002446	2.543669
GOTERM_MF_DIRECT	GO:0004252∼serine-type endopeptidase activity	5	6.28	.002603	2.705516
GOTERM_MF_DIRECT	GO:0000975∼regulatory region DNA binding	2	2.51	.025951	24.17125

KEGG analysis results are shown in Table 3, which demonstrates that DEGs were particularly upregulated in the chemokine signaling pathway, Toll-like receptor signaling pathway, and RIG-I-like receptor signaling pathway (*P* < .05).

**Table 3:** KEGG pathway analysis of differentially expressed genes in DLE.

Pathway ID	Name	Count	%	*P*-value	Genes
hsa04062	Chemokine signaling pathway	9	12.33	7.37E–06	CCR7,CXCL13,CXCL9,CCL8,CXCL11,STAT1,CCL5,STAT2,CXCL10
hsa04620	Toll-like receptor signaling pathway	7	9.59	2.51E–05	IRF7,CXCL9,CXCL11,STAT1,CCL5,TLR7,CXCL10
hsa04622	RIG-I-like receptor signaling pathway	4	5.48	.007097	IFIH1,ISG15,IRF7,CXCL10

### 3.3 PPI network and modular analysis

**Figure 2:**
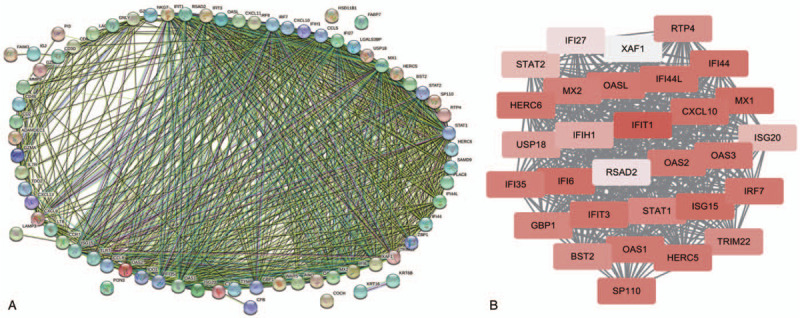
Results of PPI network analysis of Common DEGs. Circles represent genes, lines represent PPI between genes, and results inside the circle represent protein structure. Line colors represent evidence of PPI. DEGs = differentially expressed genes, PPI = protein-protein interaction.

A total of 73 DEGs were imported into the PPI network complex of 73 nodes and 717 edges, including 4 downregulated and 69 upregulated genes (Fig. 2A). To recognize the intersected clusters from the attained PPI network, we exploited the Cytoscape plugin MCODE.^[18]^ Cytotype MCODE was applied for further analysis and identified 30 central nodes, which were all upregulated genes, among the 73 nodes (Fig. 2B).

### 3.4 Re-analysis of 30 selected genes via KEGG pathway upregulation

**Figure 3:**
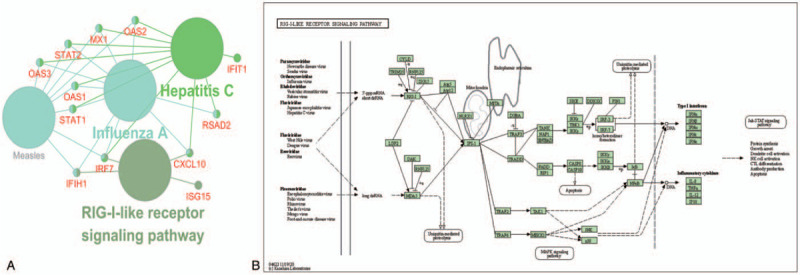
Re-analysis of 30 selected genes by KEGG pathway enrichment. A, Re-analysis of 30 selected genes via Cytoscape ClueGo. B, KEGG pathway from DAVID software. DAVID = Database for Annotation, Visualization, and Integrated Discovery, KEGG = Kyoto Encyclopedia of Genes and Genomes.

The top subnetwork from MCODE was used as an input for analyzing the possible pathway of PPI subnetworks using the ClueGO/CluePedia plugin from Cytoscape^[19]^ and Enrichr software (*P* < .05). The results showed that 4 genes (C-X-C motif chemokine ligand [CXCL]10, ISG15, IFIH1, and IRF7) were significantly upregulated in the RIG-I-like receptor signaling pathway (Table 4 and Fig. 3).

**Table 4:** Reanalysis of 30 selected genes via KEGG pathway enrichment.

Term	*P*-value	Adjusted *P*-value	Odds ratio	Combined score	Genes
RIG-I-like receptor signaling pathway	3.52E–06	1.66E–05	46.39627	582.6039	IFIH1,ISG15,IRF7,CXCL10

## 4 Discussion

Discoid lupus erythematosus (DLE), a chronic inflammatory disease, is a cutaneous manifestation of lupus that occurs more frequently in women in their 40s and 50s and, in many cases, leads to scarring, disfiguration, decreased quality of life, and increased morbidity.^[20]^ 10% to 20% of patients with SLE are diagnosed with DLE,^[21–23]^ but the molecular mechanism behind discoid lupus skin remains unclear. The current first-line treatment consists of photoprotection with topical or oral corticosteroids, topical calcineurin inhibitors, and systemic antimalarial therapy.^[24]^ Although most patients respond to this regimen, approximately 30% to 40% of cases are refractory.^[25]^ For this significant minority, there is no consensus algorithm, and a trial and error approach using multiple systemic agents has shown a variable response.^[26]^ Therefore, there is a need for new treatment strategies.

We used bioinformatics methods on 2 profile datasets, GSE52471 and GSE72535, and using GEO2R and Venn software, we found a total of 73 common DEGs (|logFC| > 2 and adjusted *P* value < .05). Gene ontology and pathway enrichment analysis using DAVID methods showed that DEGs were particularly upregulated in the chemokine signaling pathway, Toll-like receptor signaling pathway, and RIG-I-like receptor signaling pathway (*P* < .05). Next, the PPI network complex of 73 nodes and 717 edges was constructed using the STRING online database and Cytoscape software. From the PPI network complex, 30 vital upregulated genes were screened using Cytotype MCODE analysis. Finally, we reanalyzed 30 genes via Cytoscape ClueGo, Enrichr software, and DAVID for KEGG pathway amplification and found that four genes (CXCL10, ISG15, IFIH1, and IRF7) were upregulated in the RIG-I-like receptor signaling pathway (*P* < .05).

C-X-C motif chemokine ligand 10 (CXCL10), also named 10 kDa IP-10, is secreted by a cluster of differentiated (CD)4+, CD8+, natural killer (NK), and NK-T cells are dependent on Interferon (IFN)-γ, which is mediated by the interleukin (IL)-12 cytokine family.^[27]^ High levels of CXCL10 in peripheral fluids are a marker of host immune responses, especially T helper (Th)1 orientated T-cells. Recruited Th1 lymphocytes may be responsible for increased IFN-γ and tumor necrosis factor (TNF)-α production, which in turn stimulates CXCL10 secretion from a variety of cells, thereby creating an amplification feedback loop.^[28]^ Recent reports have shown that the serum and/or tissue expression of CXCL10 is increased in organ-specific autoimmune diseases such as autoimmune thyroiditis (AT), Graves disease (GD), and type 1 diabetes (T1D), or in systemic rheumatological disorders such as rheumatoid arthritis (RA), systemic lupus erythematosus (SLE), systemic sclerosis (SSc), and cryoglobulinemia.^[28,29]^ Narum et al^[30]^ reported increased serum levels of CXCL10 in patients with SLE, which strongly correlated with disease activity. Another study on plasma chemokine concentrations in SLE showed that plasma concentrations and ex vivo mitogen-induced peripheral blood mononuclear cell production of CXCL10 and MCP-1 (CCL2) were increased in SLE patients.^[31]^ Other studies have also shown a strong, positive correlation between CXCL10 levels and SLE disease activity and suggested a possible correlation between renal involvement in SLE and CXCL10.^[32,33]^ Although there is evidence that CXCL10 levels are elevated in sera and/or tissues of SLE patients, the exact role of CXCL10 in the pathogenesis of SLE remains to be elucidated.

Interferon-stimulated gene 15 (ISG15), a type I IFN-dependent transcript, is a member of the ISG protein family that encodes a ubiquitin-like protein.^[34,35]^ ISG15 has complex biological links, on the one hand, to the covalent intracellular modification of target proteins (ISGylation, a process similar to ubiquitinylation) and, on the other hand, plays a role as an extracellular signaling molecule/cytokine.^[36]^ ISG15 is thought to play a critical role in innate immune responses, the progression of immune diseases, and anti-tumor reactions.^[37]^

Interferon-induced helicase C domain 1 (IFIH1), encoding MDA5, is associated with autoimmune diseases. A genome-wide association study (GWAS) revealed that SNPs of IFIH1 are significantly associated with the risk of autoimmune diseases such as T1D, MS, psoriasis, selective IgA deficiency, and SLE.^[38–42]^

Interferon regulatory factor 7 (IRF7) belongs to the IRF family, which is a key player in multiple facets of host defense systems.^[43,44]^ The IRF7 gene was originally cloned in 1997^[45]^ and is a key regulator of the type I IFN (IFNα/β) response, which is central to both innate and adaptive immunity.^[46]^ As IRF7 is also activated by TLR3/7/9 and RIG-I in response to nucleic acids, and pDCs, in which the TLR7/IRF7 pathway governs type I IFN production, secrete a large amount of IFNα in response to immune complexes, and has a role in autoimmune diseases, IRF7 may also be involved in autoimmune diseases.^[46–48]^ A recent study of single-nucleotide polymorphisms in the Irf7/Phrf1 locus has also indicated that genetic variants of Irf7 act as risk factors for SLE, but the roles of these variants require further investigation.^[49]^ Wu et al^[50]^ indicated that the upregulation and activation of IRF7 in SSc skin plays an important role in the observed exaggerated inflammation, as well as fibrosis, and may provide a link between the prominent IFN signature and activation of TGF-β in SSc. Fu et al^[51]^ demonstrated that the functional IRF7 variant rs1131665 (Q412R) was associated with SLE, and Heinig et al^[52]^ reported that IRF7 was implicated in the pathogenesis of type I diabetes.

## 5 Conclusion

In summary, our results, based on 2 different microarray datasets taken from normal skin and DLE tissue samples, reinforce the notion that 4 DEGs (CXCL10, ISG15, IFIH1, and IRF7) could play crucial roles in the pathophysiological mechanisms of DLE. However, these predictions should be verified through a series of future experiments. Regardless, these data may provide useful information about new genes linked to DLE pathogenesis with a view to better understand its development to develop new treatments against it.

## Author contributions

**Data curation:** Yanping Feng, Jianqiang Lu.

**Formal analysis:** Qian Dong, Hui Han.

**Methodology:** Qian Dong.

**Writing – original draft:** Qian Dong, Kang Chen.

**Writing – review & editing:** Qian Dong, Kang Chen, Jinye Xie, Weijia Wang.
